# Characterization of Haplotype Diversity in the *BADH2* Aroma Gene and Development of a KASP SNP Assay for Predicting Aroma in U.S. Rice

**DOI:** 10.1186/s12284-020-00410-7

**Published:** 2020-07-14

**Authors:** Christopher K. Addison, Brijesh Angira, Manoch Kongchum, Dustin L. Harrell, Niranjan Baisakh, Steven D. Linscombe, Adam N. Famoso

**Affiliations:** 1 School of Plant, Environmental and Soil Science, Louisiana State University, 104 Sturgis Hall, Baton Rouge, LA 70803 USA; 2 H. Rouse Caffey Rice Research Station, Louisiana State University Agricultural Center, 1373 Caffey Rd, Rayne, LA 70578 USA

**Keywords:** Rice, Aroma, Haplotype analysis, BADH2, SNP

## Abstract

**Background:**

Aroma is an important grain quality trait in rice, controlled by mutations within the *BADH2* gene. The trait is simply inherited, and its importance in variety development makes it a practical target for marker-assisted selection in applied breeding programs. The predominant functional mutation within *BADH2,* an 8-bp indel, can be reliably detected using a PCR-based assay, but the available assays and associated genotyping platforms are insufficient for large-scale applied molecular breeding applications and are not compatible with outsourcing genotyping services.

**Results:**

We first characterized SNP diversity across the *BADH2* gene in a collection of 2932 rice varieties to determine the number of gene haplotypes in *O. sativa*. Using 297 gene-based SNPs, 11 haplotype groups were detected, and subsequently identified a minimal set of nine informative SNPs that uniquely identified the *BADH2* haplotypes. These nine SNPs were developed into KASP assays and used to examine a panel of 369 U.S. rice accessions. The panel represented modern breeding germplasm and included all known aroma pedigree sources in U.S. rice. Six haplotypes were detected within the U.S. panel, of which two were found in majority (85%) of varieties. A representative set of 39 varieties from all haplotype groups was evaluated phenotypically to distinguish aromatic from non-aromatic lines.

**Conclusion:**

One haplotype (Hap 6) was found to be perfectly associated with the aromatic phenotype. A single KASP SNP unique to Hap 6 was demonstrated to reliably differentiate aromatic from non-aromatic rice varieties across U.S. germplasm.

## Background

Rice is a staple crop that provides the largest amount of daily caloric intake for human populations globally. Most rice is consumed within the country where it was produced, with only 7% of rice production entering the global export market (Giraud, 2013; Muthayya et al., 2014). Aromatic varieties account for a significant portion of the export market and include widely recognized types, including Jasmine and Basmati rice. These two types account for the majority of the aromatic rice global production (Annex, 2011; Giraud, 2013; Mahajan et al., 2018). The USDA forecasts imports of rice for the United States to be 27 million hundredweight (cwt) in 2018, with 23.5 million cwt being aromatic, long-grain varieties classified as either Jasmine or Basmati types (Baldwin and Childs, 2018).

The genetic relationship between fragrant rice varieties and other subpopulations of rice has become clearer with the use of molecular markers. Rice (*Oryza sativa*) was domesticated roughly 10,000 years ago from pre-differentiated gene pools of the wild grass species *Oryza rufipogon* (Kovach et al., 2007). These gene pools, *Indica* and *Japonica*, are hypothesized to have diverged between 200,000–400,000 years prior to their domestication (Cai and Morishima, 2002; Ma and Bennetzen, 2004; Vitte et al., 2004). Several studies have identified five genetically distinct subpopulations within these varietal groups. The *Indica* varietal group contains the *indica* and *aus* subpopulations, and the *Japonica* varietal group contains the *temperate japonica, tropical japonica,* and *aromatic* subpopulations (Glaszmann, 1987; Garris et al., 2005; Kovach et al., 2007; Civán et al., 2015; McCouch et al., 2016). The *aromatic* subpopulation was previously assumed to be most closely related to the *indica* types based on the morphological similarity of the grain, but research utilizing simple sequence repeats demonstrated that the *aromatic* subpopulation was genetically more closely related to the *japonica* than the *indica* subpopulation (Sweeney and McCouch, 2007). Today, based on re-sequencing studies, the *aromatic* subpopulation is interpreted to be an ancient admixture between *temperate japonica* and *aus,* with a minor dose of *indica* ancestry (Civán et al., 2015). In the United States, most rice germplasm belongs to the *Japonica* varietal group, with long-grain germplasm associated with the *tropical japonica* subpopulation and medium-grain germplasm containing both *tropical* and *temperate japonica* ancestry (Lu et al., 2005; Zhao et al., 2011).

Thirteen fragrant rice varieties have been developed in the United States, with the fragrance trait derived from six different aromatic varieties that were introduced into U.S. breeding germplasm. The introduced sources of aroma include Delitus, Basmati 370, Jasmine 85, 96A-8, and two additional Basmati types (Linscombe and Famoso, 2017; McClung, 2018; Marchetti et al., 1998; Sha et al., 2011; CCRRF, 2018).

The method for detecting fragrance in rice was initially scent-based, involving KOH and ground leaf or stem tissue (Sood and Siddiq, 1978). The component, 2-acetyl-1-pyrroline (2AP), was subsequently identified as the key factor of the aromatic qualities present in fragrant rice varieties (Buttery et al., 1982), and a method was developed for quantifying 2AP with gas chromatography (GC) (Petrov et al., 1996). A strong correlation was observed between the GC method and the standard human scent technique, with the GC method demonstrating less subjectivity and increased accuracy (Lorieux et al., 1996). Although GC is a reliable method, the throughput is relatively low, and it is cost prohibitive in the context of an applied breeding program where large sample numbers are typical.

A major gene (*fgr)* was detected in multiple studies for 2AP content. This gene was reported on Chromosome 8 and explained up to 69% of the variation for 2AP content (Ahn et al., 1992; Lorieux et al., 1996; Chen et al., 2006). The underlying gene is a betaine aldehyde dehydrogenase gene (*BADH2*), and the functional mutation was demonstrated to be an 8-base pair (bp) deletion located in the seventh exon of the gene (Bradbury et al., 2005a). The proposed pathway of 2AP production begins with proline being catabolized via putrescine into y-aminobutyraldehyde (AB-ald), which is a substrate of *BADH2*. A functioning *BADH2* enzyme converts AB-ald into y-aminobutyric acid (GABA). Fragrant rice lacks a functioning *BADH2* enzyme which causes an accumulation of AB-ald. Due to the inability of the enzyme to convert AB-ald into GABA, an increased synthesis of 2AP results from the accumulated AB-ald being acetylated (Bradbury et al., 2008; Chen et al., 2008). Although 10 different alleles have been reported that appear to confer fragrance, the 8-bp deletion is the most predominant fragrance allele. The 8-bp insertion/deletion (indel) was present in 93/124 (80%) diverse fragrant varieties from around the world, including common fragrant varieties, such as KDML105, Basmati, Della, and Jasmine 85 (Kovach et al., 2009). The discovery of the 8-bp functional deletion facilitated the development of an indel DNA marker for use in research and breeding applications (Bradbury et al., 2005a, b).

The fact that a single gene controls a large proportion of the phenotypic variation for aroma and that the cost and throughput of the phenotyping is a limitation in the breeding of new aromatic lines, makes this an ideal trait for marker-assisted selection. With an increasing interest in the development of fragrant varieties for U.S. rice production, the availability of a low-cost, high-throughput, single nucleotide polymorphism (SNP)-based DNA marker suitable for screening U.S. breeding material for fragrance and compatible with commonly used KASP genotyping platforms would be of immediate utility for applied breeding programs. The objectives of this research were to 1) characterize the haplotype diversity of the *BADH2* gene across *O. sativa*, 2) determine which *BADH2* haplotypes/alleles are present in U.S. rice germplasm, and 3) develop and validate a SNP-based kompetitive allele specific polymerase chain reaction (KASP) assay (LGC Group, 2016) informative across U.S. breeding germplasm and useful for high-throughput genotypic selection for fragrance in U.S. breeding programs.

## Materials and Methods

### Haplotype Variation in *BADH2* in the 3 K Genomes Dataset

Single nucleotide polymorphism variation across the *BADH2* (LOC_Os08g32870) gene was obtained from the International Rice Informatics Consortium (IRIC) SNP-Seek database, from the “3kAll” SNP dataset (http://snp-seek.irri.org/) (Mansueto et al., 2017). Single nucleotide polymorphism data points with heterozygous allele calls were classified as missing data, and SNPs with > 100 total missing data points were excluded from haplotype characterization. The graphical genotyping and haplotype visualization software Flapjack (Milne et al., 2010) was used for initial haplotype grouping and characterization. Haplotypes were defined based on a SNP similarity of > 0.99, with genotypes having three or less SNPs different being grouped as a haplotype class. Haplotypes that were present in less than 5% of the genotypes were defined as rare and subsequently omitted from haplotype analysis. Haplotype groups that contained genotypes that differed by three or less SNPs were further divided into subgroups. Subgroups were defined by all genotypes having identical SNP data and the haplotype being present at a minimal frequency of 1% of the total genotypes within the haplotype group. These haplotype subgroups were designated with an A, B, or C. If a subgroup did not consist of 1% of the total number of haplotypes for the subpopulation, it was considered extremely rare and designated with an “X” (Supplemental Table 1). Genotypes that had missing data for the key SNPs and could not be accurately classified were designated as unclassified (U).

### U.S. Rice Germplasm Panel

The U.S. rice germplasm panel consists of 369 accessions that represent all modern southern U.S. rice breeding germplasm, including modern varieties, parental lines, and advanced breeding materials. The panel includes 27 aromatic lines and offspring derived from pedigrees that include all known aromatic sources used in U.S. breeding. The panel consists of germplasm from Louisiana (131), Arkansas (93), Texas (74), Mississippi (44), and California (8). The international diversity is comprised of germplasm from Italy, Japan, Brazil, Taiwan, and Uruguay. The panel includes short- (7), medium- (45), and long-grain (289) rice accessions as well as a collection of herbicide resistance classes.

### Minimum SNP Set

To identify a minimal subset of SNPs that could unambiguously differentiate the *BADH2* haplotypes found in the 3 K genomes, the 255 SNPs found within the *BADH2* gene were analyzed across the 11 haplotypes present in the 3 K accessions. The SNPs were sorted by minor allele frequency among the 11 haplotype groups. Each SNP was selected one at a time to distinguish the haplotype groups. Upon each SNP selection, the haplotype groups were further distinguished, and the subsequent SNP selection was based on the SNPs capacity to distinguish any haplotype groups that were yet to be differentiated by the minimum SNP set. A set of 8 SNPs (designated SNP2 – SNP9) was identified that could successfully distinguish all of the haplotypes, with the exception of Hap 7 and Hap 9, rare *aus* haplotypes which could not be distinguished. We next sought to identify a single SNP that would be diagnostic of Haplotype 6 (Hap 6), the haplotype carried by majority of lines known to be fragrant. None of the SNPs within the *BADH2* gene met this criterion, so the 10-kb sequence on either side of the *BADH2* gene were examined to identify a SNP (designated SNP 1) that would uniquely distinguish Hap 6.

### KASP Assay Development and Genotyping

Kompetative allele specific polymerase chain reaction assays were designed through LGC genomics (LGC Group, 2016) to characterize the nine SNPs used to differentiate *BADH2* haplotype groups (described above). An additional KASP assay was designed to characterize the previously reported functional 8-bp deletion in the *BADH2* gene (Bradbury et al., 2005a, b). All KASP assay genotyping was performed using the LGC SNPline system following standard KASP protocols (LGC Group, 2016) at the H. Rouse Caffey Rice Research Station (HRCRRS). Leaf tissue was collected from the flag leaf of field-grown plants at harvest and dried at 38 °C for 3 days. DNA was extracted using a modified CTAB method for KASP genotyping (Khan et al., 2013).

The trait marker, SNP1, was additionally designed into an assay by 3CRbio (www.3crbio.com) with the primer sequences:

Allele 1: GAAGGTGACCAAGTTCATGCTTGGTAGCAATCTAAATAGGCACGTAA,

Allele 2: GAAGGTCGGAGTCAACGGATTGGTAGCAATCTAAATAGGCACGTAC, Common: TACTTGTATATACTTGCAGCCATGAATGTT.

The functional 8 bp deletion was assayed using a gel based indel marker as described by Bradbury et al., 2005b. PCR was performed as described by Solis et al., 2018, with minor modifications. Briefly, 50 ng of genomic DNA was amplified in a total volume of 20 ul that contained 2 ul of 10x PCR buffer, 2.5 ul of 25 mM MgCl2, 2.5 ul of 2 mM dNTP mix, 0.5 ul of 10 uM primers, and 0.2 ul of Taq polymerase (Promega, Madison, WI). The thermal profile was initial denaturation for 5 min at 94 °C, followed by 35 cycles of denaturation at 94 °C for 30 s, annealing at 58 °C for 30 s, elongation at 72 °C for 45 s, and a final extension at 72 °C for 5 min. The PCR products were resolved in 1.5% TAE-agarose gel and viewed/captured in GelLogic 200 imaging station (Kodak Inc., New Haven, CT).

### Analysis of Fragrance

A subset of 39 varieties was selected from the U.S. germplasm panel and phenotyped for fragrance. These varieties represented all six of the haplotype classes observed in the U.S. germplasm, and 27 fragrant rice varieties that represented all known pedigree sources of aroma in U.S. germplasm. Seeds were obtained from panicle rows grown at the HRCRRS in 2017. Five grams of milled rice of each line were prepared by grinding them into a powder of less than 2.5 mm in diameter with a Cyclone Sample Mill (UDY Corporation). The 2AP concentration was determined using gas chromatographic separation on a Shimadzu GC-2010 *Plus* System (Shimadzu, Columbia, MD) with a flame thermionic detector (FTD) following a modified method of the protocol described by Goufo et al., 2010. Pressuring time, pressure equilibrium time, and injection times were set at 1, 0.01, and 2 min, respectively. The headspace was transferred to the gas chromatograph with a heat transfer line for 0.5 min. Oven, sample line, and transfer line temperatures were set at 120, 150, and 160 °C, respectively. Gas chromatography and FTD were conducted with the temperatures of the detector set to 280 °C. Helium was used as the carrier gas with a flow rate of 3.5 mL/min. Data were collected on three replications per sample using LabSolutions software (Shimadzu, Columbia, MD), and the average value was assigned for the 2AP concentration.

## Results

### *BADH2* Sequence and Haplotype Diversity in *O. sativa*

To characterize the extent of sequence variation across the *BADH2* gene, we examined the SNP-Seek database containing re-sequencing information for a collection of 2932 (hereafter referred to as 3 K) diverse varieties of *O. sativa* (http://snp-seek.irri.org). A total of 297 SNPs was identified in the *BADH2* (LOC_Os08g32870) gene. Filtering for heterozygous calls and missing data eliminated 42 SNPs, leaving 255 SNPs that were utilized for construction of *BADH2* gene haplotypes. The number of SNPs observed at the *BADH2* gene in the SNP-Seek database was similar to previously reported genes (Wang et al., 2018). Eleven haplotype groups were identified and summarized in Supplemental Table 1. All but 143 accessions (4.8%) could be classified into one of the 11 haplotype groups. Accessions that could not be classified included 74 that had rare haplotypes (present at a frequency of 2.5%), and 69 that were unclassified due to missing data (> 100 data points missing).

Haplotype 1 (Hap 1) was the most common haplotype in the dataset, present in 892 (30%) accessions. It was the most frequent haplotype in *indica* (42%; *n* = 749), and the second most frequent haplotype in the *tropical japonica* subpopulation (26%; *n* = 121) (Table 1). Haplotype 2 (Hap 2) was the second most common haplotype in the 3 K genomes dataset, present in 755 (26%) accessions. It was the predominant haplotype within the *Japonica* varietal group (70%; *n* = 586), present in 96% of *temperate japonica* and in 60% of *tropical japonica* accessions (Table 1).

**Table 1:** Haplotype characterization of the *BADH2* gene across the global rice diversity panel

	Subpopulation Ancestry^b^								20374951^a^	20,381,787	20,382,161	20,384,350	20,382,286	20,380,447	20,381,308	20,380,886	20,382,480
									SNP1^c^	SNP2	SNP3	SNP4	SNP5	SNP6	SNP7	SNP8	SNP9
**Hap Group**	**Aus**	**Indica**	**Admix**^d^	**ARO.**	**TEJ.**	**TRJ.**	**Japx**	**Total**	**601**^e^	**548**	**536**	**603**	**531**	**849**	**852**	**855**	**N/A**
1	1	749	12	0	1	121	8	892	G	A	G	G	A	G	G	T	C
2	2	148	19	26	215	281	64	755	G	T	A	G	A	C	G	T	C
3	0	201	1	0	1	0	0	203	G	A	G	A	A	C	C	T	C
4	9	183	5	0	0	0	0	197	G	A	G	G	A	G	C	T	C
5	1	186	5	2	0	1	0	195	G	T	G	G	A	C	C	T	C
6 (aromatic)	0	65	9	38	3	53	3	171	T	T	A	G	A	C	C	T	C
7	97	11	6	0	0	0	0	114	G	A	G	G	A	C	C	T	C
8	0	89	2	1	0	2	0	94	G	A	G	G	G	G	G	T	C
9	63	13	6	3	0	0	0	85	G	A	G	G	A	C	C	T	C
10	1	39	6	0	2	4	0	52	G	A	G	G	A	G	G	T	T
11	3	22	6	0	0	0	0	31	G	A	G	G	A	G	C	C	C
R	22	42	6	2	0	2	0	74	–	–	–	–	–	–	–	–	–
U	2	41	16	3	2	3	2	69	–	–	–	–	–	–	–	–	–
Total	201	1789	99	75	224	467	77	2932									

^a^SNP positions are based on IRGSPv.1

^b^Subpopulation assignments based on SNP-Seek assignments, with all Indica subgroups classified as Indica

^c^Represents the SNP ID used throughout manuscript

^d^Admix, *admixture*; ARO, *aromatic*; TEJ, *temperate japonica*; TRJ, *tropical japonica*; Japx*, japonica admixture*

^e^Represents the LSU KASP SNP ID. SNP9 was not developed into a KASP assay

Haplotypes 3, 4, 5, 7, 8, 9, 10, and 11 were predominantly carried by *indica* or *aus* accessions, as summarized in Table 1. These haplotypes were relatively minor (0.7–7%) in the dataset as a whole; however, they are consistent with observations that there is greater genetic variation in *indica* and *aus* than in the *tropical japonica* and *temperate japonica* subpopulations of *O. sativa* (Kovach et al., 2009; Zhao et al., 2011; Huang et al., 2012; McCouch et al., 2016).

Haplotype 6 (Hap 6) was rare in the 3 K genomes (5.8%; *n* = 171) overall, but it was the most common haplotype within accessions belonging to the *aromatic* subpopulation, present in 51% (*n* = 38) of accessions in the subpopulation. Hap 6 was absent in *aus* and found at relatively low frequencies in the other subpopulations (1.3% in *temperate japonica*, 3.6% in *indica*, 11% in *tropical japonica*). The *aromatic* subpopulation only accounts for 2.6% (75 accessions) in the 3 K genomes dataset. Due to the relatively lower representation, there is a bias in the number of Hap 6 containing accessions across subpopulations in this study. For example, of the 171 accessions containing Hap 6, 22% were from the *aromatic* subpopulation, 38% were from the *indica* subpopulation, and 31% were from the *tropical japonica* subpopulation (Table 1). This observation highlights the fact that some accessions do contain the aroma allele at *BADH2*, while belonging to another subpopulation on the whole genome level.

Known fragrant accessions, such as Domsiah, Basmati 1, Gerdeh, and the Thai Jasmine variety, Khao Dawk Mali 105, all carried Hap 6, and all had been documented to contain the functional 8-bp deletion causing fragrance in rice (Mahatheeranont et al., 2001; Bradbury et al., 2005a; Kovach et al., 2009). Based on these observations, it was hypothesized that Hap 6 carries the functional 8-bp deletion that is responsible for the fragrant phenotype, and that all Hap 6-containing accessions would be phenotypically fragrant.

To confirm this hypothesis, we first interrogated the SNP-Seek database to find accessions carrying the 8-bp deletion. The deletion was observed in 128 of the 3 K accessions, all of which were classified as carrying Hap 6, but interestingly, 43 Hap 6-containing accessions in SNP-Seek did not appear to carry the deletion. Among these accessions were two well-known accessions, Khao Dawk Mali 105 and Nerica 1 which are known to be fragrant. Previous studies had clearly documented that these two fragrant varieties did, in fact, carry the 8-bp deletion (Kovach et al., 2009; Asante et al., 2010). This observation suggested that the indel data from the SNP-Seek database was not detecting the 8-bp deletion in some samples or that different seed sources of these accessions were used across different studies.

### Development of KASP Assays for Haplotype Characterization

**Fig. 1:**
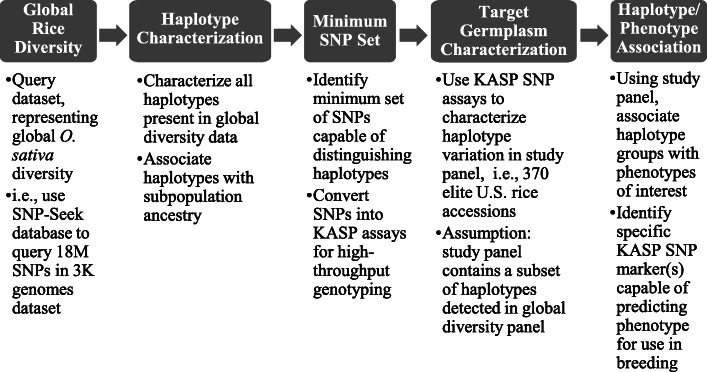
Haplotype Characterization and Phenotypic Validation Workflow. Legend: Workflow utilized to leverage publicly available genomic information to develop a KASP SNP marker for targeted application in U.S. rice breeding germplasm

To attempt to resolve the discrepancy, attention was turned to the development of KASP assays that could reliably identify the major *BADH2* haplotypes, with particular attention on a rapid and cost-effective assay that could diagnose Hap 6 and tag the functional 8-bp deletion causing fragrance. A minimal set of 8 SNPs (designated SNP2 – SNP9), selected from the 255 SNPs segregating in the *BADH2* gene, was selected that could unequivocally identify all major haplotypes, with the exception of Hap 9 which could not be distinguished from Hap 7 (Fig. 1). These two, relatively rare haplotype groups were primarily found in the *aus* subpopulation (Table 1) and given the emphasis on U.S. germplasm in our study, differentiating haplotypes 7 and 9 was not considered a priority.

Next, a single SNP that would be uniquely diagnostic of Hap 6, the haplotype carried by majority of lines known to be fragrant, was sought to be identified. None of the SNPs within the *BADH2* gene met this criterion, so the 10-kb sequence upstream and downstream of the *BADH2* gene was examined. A Hap 6-diagnostic SNP, designated SNP 1, was identified 7908 bp from the *BADH2* gene. This SNP is invariant in all the other haplotypes (Fig. 1).

### *BADH2* Diversity in U.S. Breeding Germplasm

The 3 K genomes dataset included only 32 varieties from the United States and did not include any modern-day varieties or breeding germplasm. Therefore, to characterize the haplotype diversity present in modern U.S. breeding germplasm, a panel of 369 lines representing modern varieties and elite breeding germplasm from the southern United States was assembled, including 27 accessions classified as aromatic based on variety/breeder description (Supplemental Table 2). This panel was characterized for haplotype diversity using the SNP-based KASP assays described above.

Eight of the nine SNPs selected for KASP assay development were successfully converted into KASP assays and used to screen the U.S. panel. SNP9, which was initially included to differentiate Hap 1 from a rare haplotype, Hap 10, in the 3 K genomes dataset, failed to convert and was omitted from further analysis. Hap 10 was observed in only 52 genotypes in the 3 K genomes dataset, primarily of *indica* origin from South and Southeast Asia, and was considered unlikely to be present in the U.S. germplasm; thus, for the purposes of this study, U.S. genotypes characterized as having Hap 1 or Hap 10 were classified as Hap 1.

In addition, a KASP assay was designed to directly characterize the 8-bp functional deletion in the *BADH2* gene. This assay accurately classified all non-deletion, non-aromatic genotypes as such, but it was unreliable for classifying accessions carrying the deletion. Indeed, 50% of known fragrant U.S. varieties were misclassified into the non-fragrant group using this KASP marker, a problem that was similar to that observed in the SNP-Seek database. Given that the U.S. panel contained experimentally confirmed fragrant and non-fragrant phenotypes, had well-documented pedigrees, and that all donor varieties carried Hap 6 and the 8-bp deletion causing fragrance, it was concluded that the KASP assay was unreliable, and therefore, all subsequent analysis focused on haplotype classification using the eight reliable SNPs (Table 2).

**Table 2:** Haplotype characterization and 2AP concentration across U.S. breeding germplasm panel

					20374951^a^	20,381,787	20,382,161	20,384,350	20,382,286	20,380,447	20,381,308	20,380,886
		2AP Concentration Test (ppm)^b^			SNP1^c^	SNP2	SNP3	SNP4	SNP5	SNP6	SNP7	SNP8
**Hap Group**	**Number**	**n**	**Range**	**Mean (SE)**^d^	**601**^e^	**548**	**536**	**603**	**531**	**849**	**852**	**855**
1	181	5	0–0	0	G	A	G	G	A	G	G	T
2	134	9	0–0.079	0.009 (0.005)	G	T	A	G	A	C	C	T
3	11	2	0–0	0	G	A	G	A	A	C	C	T
5	5	3	0–0.001	0.0002 (0.0002)	G	T	G	G	A	C	C	T
6 (aromatic)	27	17	0.356–3.165	1.30 (0.058)	T	T	A	G	A	C	C	T
7 or 9	11	3	0–0.009	0.003 (0.002)	G	A	G	G	A	C	C	T

^a^SNP positions are based on IRGSPv.1 and are on Chromosome 8

^b^ppm, parts per million

^c^Represents the SNP ID used throughout manuscript

^d^Values in the parentheses represent the standard error of the 2AP Concentration

^e^Represents the LSU KASP SNP ID

Of the 11 haplotype groups present in the 3 K genomes dataset, six were identified in the U.S. germplasm, with Hap 1 (49%, *n* = 181) and Hap 2 (36%, *n* = 134) cumulatively accounting for 85% of the panel (Table 2). Accessions carrying Hap 1 were predominantly (92%) long-grain varieties, but it was also found in 14 medium-grain and one short-grain variety. Hap 2 was most common in medium-grain varieties, present in 67% of the 45 medium-grain accessions in the U.S. panel. Given that long-grain U.S. varieties are primarily classified as *tropical japonicas,* and medium-grain varieties as *tropical* and *temperate japonica,* the frequencies of Hap 1 and Hap 2 in the U.S. panel is consistent with observations of haplotype frequencies in the 3 K genomes dataset.

Hap 6 was observed in 27 accessions in the U.S. germplasm panel and 17 of these lines were selected as a subset for phenotyping for 2AP content. All 17 lines that contained Hap 6 were phenotypically characterized as fragrant. This is consistent with the hypothesis that Hap 6 carries the 8-bp deletion in the *BADH2* gene, and that fragrance in southern U.S. germplasm is perfectly predicted by the presence of Hap 6. Thus it was concluded that fragrant U.S. germplasm contains the same *BADH2* allele as identified in the Thai Jasmine line KDML105 (Kovach et al., 2009).

Three haplotypes (Hap 3, Hap 5, and Hap 7/9) strongly associated with the *Indica* varietal group in the 3 K genomes dataset were observed in a small set of U.S. varieties (Table 2). Hap 3 was identified in 11 breeding lines, 10 of which shared historical pedigree relationships that traced back to the Texas variety ‘Dawn’ (Supplemental Table 2) (Bollich et al., 1968). The key intermediates between Dawn and the 11 modern breeding lines include ‘Trenasse’ and ‘Catahoula’ (Linscombe et al., 2006; Blanche et al., 2009). It was concluded that, in these breeding lines, Hap 3 derives from Dawn, which inherited the *BADH2* gene from an *indica* ancestor. The one additional U.S. line containing Hap 3 was ‘CL151’, which has the *indica* variety ‘Taducan’ in its pedigree and is the likely source of Hap 3 in that lineage (Blanche et al., 2011). Hap 5 was observed in five U.S. lines, two of which are recent introductions from outside the United States, and two are new Provisia herbicide-resistant breeding lines derived from *indica* breeding germplasm. Hap 7/9 is present in 11 U.S. varieties, eight of which were developed in Arkansas and share similar pedigree histories that trace back to the introduction of ‘Rexoro’ (Johnston, 1958).

To confirm the presence or absence of fragrance in the U.S. panel, a representative sample of each haplotype group was phenotyped for 2AP content. A set of 39 accessions was phenotyped using gas chromatographic separation to quantify 2AP content in milled rice samples (Table 2). Seventeen accessions that contained Hap 6 and had been previously characterized as fragrant varieties based on breeder descriptions were phenotyped and all were detected to contain 2AP. The average 2AP content for Hap 6-containing varieties was 1.30 ppm and ranged from 0.356 to 3.165 ppm. The range of measured 2AP content in fragrant genotypes demonstrates that additional, small-effect genes are involved in determining the level of 2AP production. A set of 22 varieties from the U.S. panel, representing all of the other haplotype classes, contained 2AP levels at or near zero, demonstrating that Hap 6 is the only aromatic haplotype in the U.S. rice panel. Based on these findings, it was concluded that the *BADH2* allele referred to in this study as Hap 6, is the only allele conferring fragrance in U.S. germplasm.

### KASP Assay Validation for Use as BADH2 Trait Marker

The haplotype SNP (SNP1) located at 20,374,951 MB is capable of differentiating the aromatic Hap 6 from all other non-aromatic haplotypes and hypothesized to be in linkage disequilibrium (LD) with the functional 8 bp deletion. The U.S. rice germplasm panel was screened with the previously reported gel-based marker that directly assays the 8 bp deletion in order to confirm the SNP is in LD with the indel (Bradbury et al., 2005b). The allele calls observed with the functional indel marker matched perfectly with the SNP1 KASP allele calls, demonstrating that the SNP1 KASP assay is in perfect LD across the U.S. germplasm panel (Supplemental Table 2).

The technical performance of the SNP1 KASP assay was evaluated in three separate genotyping jobs, run across 351 lines from the U.S. germplasm panel. The data return across the 1053 reactions was 99%., with 11 reactions resulting in missing data. Seven reactions failed to amplify and four reactions resulted in unclear clustering, where the data point clustered between the heterozygous and homozygous G clusters. The reproducibility across the three genotyping jobs was 100%. A segregating population of 96 F2:F3 plants from a bi-parental breeding population was evaluated to investigate the clustering of the assay and the potential for auto-scoring in the presence of the heterozygous class. The SNP1 KASP assay produced clearly defined clusters and was capable of autoscoring, thereby making this assay amenable to high-throughput KASP genotyping for breeding applications (Supplemental Figure 1). Based on these validation procedure, we conclude that the KASP assay of SNP1 (Chr 8: 20,374,951 bp) is capable of differentiating aromatic from non-aromatic lines across U.S. rice breeding germplasm and is suitable for high-throughput breeding applications.

## Discussion and Conclusions

The objective of this research was to leverage publicly available, high density, SNP data from *O. sativa* to develop a rapid, cost-effective DNA marker assay for use in an applied U.S. rice breeding program. A common hurdle in the utilization of public genomic resources for applied breeding objectives is that elite germplasm of interest to plant breeders often is not well represented. A valuable feature of the 3 K genomes SNP-Seek database is that it represents the global diversity of domesticated Asian rice (*O. sativa*). It is reasonable to assume that haplotype alleles present in a specific rice breeding program are represented within the 3 K genomes, even if the specific accessions are not. In this study, the 3 K genotype data was utilized to characterize, identify, and validate that the *BADH2* allele that confers fragrance in U.S. rice germplasm, without any fragrant U.S. varieties being present in the 3 K genomes dataset. By characterizing the haplotype diversity found in the 3 K genomes using 255 genic SNPs, a subset of nine SNPs was identified that together could distinguish all 11 haplotypes, effectively obtaining the same resolution as with the full 255-SNP set. Hap 6 was determined to be the aromatic haplotype due to 128 lines within the haplotype containing the 8-bp deletion.

Then a panel of U.S. germplasm was genotyped using the nine SNPs to characterize *BADH2* gene diversity in this panel. This set of nine SNPs could be applied in a similar fashion to other breeding programs’ target germplasm, assuming the haplotypes present in the target germplasm were represented in the 3 K genomes dataset. The panel of U.S. germplasm included all modern U.S. varieties and elite breeding lines, thereby representing the pool of potential parents of new breeding crosses. In addition, the panel included fragrant lines whose pedigrees traced back to all original sources of fragrance used in U.S. rice breeding. Thus, the aroma trait SNP (SNP1) validated across this panel can be used with confidence in any breeding crosses that are derived from materials in the panel.

Although this SNP has been validated across the target U.S. breeding germplasm, it should be noted that the nature of LD based markers is germplasm dependent. The aroma SNP (SNP1) identified in this work was in perfect LD across the target germplasm; however, it is very possible that this SNP will not be in LD with the 8 bp deletion across all germplasm globally. Thus, as with any LD based marker, validation in the target breeding germplasm is necessary prior to deployment for breeding applications. Similarly, attempts in this work to develop a KASP assay on the 8 bp deletion were not successful when test on U.S. japonica rice, thus it is possible that the KASP assay could be successfully utilized on *indica* germplasm or a different assay design could provide better results.

The ultimate objective of this research was to identify a single SNP that could be used for selection of fragrant varieties within U.S. breeding programs. For deployment in an applied breeding program, a DNA marker must be accurate across the target germplasm, amenable to high-throughput genotyping, and effective on low cost genotyping platforms/chemistries (Platten et al., 2019). Many breeding programs utilize KASP genotyping chemistry (LGC Group, 2016), which is ideal for SNP assays but can also detect small indels. In this study, the development of a KASP assay targeting the causal 8-bp deletion for fragrance was attempted but had poor and inconsistent genotyping results. It is common that a functional mutation would involve an indel, and in cases where developing a robust KASP assay on the indel itself is unsuccessful, the haplotype approach described in this study can be effectively utilized to identify a SNP in linkage disequilibrium with the functional polymorphism.

## Supplementary information

**Additional file 1: Supplemental Table 1**. *BADH2* SNP output and haplotype assignments from 3 K genomes data set. **Supplemental Table 2**. Haplotype characterization of the U.S. breeding germplasm panel using the minimal SNP set.

**Additional file 2: Supplemental Figure 1**. Technical validation of Aroma SNP1 across segregating F2:F3 breeding population. All genotypic classes showed clear clustering suitable for automated computer scoring with the exception of one line (indicated in grey).

## Data Availability

Datasets supporting the results are included as supplemental files.

## Abbreviations

Hap: Haplotype
KDML105: Khao Dawk Mali 105
*BADH2*: Betaine aldehyde dehydrogenase gene
KASP: Kompetitive allele specific polymerase chain reaction
SNP: Single nucleotide polymorphism
bp: Base pair
2AP: 2-acetyl-1-pyrroline
SSRs: Simple sequence repeats
GC: Gas chromatography
AB-ald: y-aminobutyraldehyde
GABA: y-aminobutyric acid
indel: insertion/deletion
FTD: Flame thermionic detector
