# Supplementary material for: Characterization of Haplotype Diversity in the BADH2 Aroma Gene and Development of a KASP SNP Assay for Predicting Aroma in U.S. Rice
Source: Rice (N Y). 2020 Jul 14;13:47. doi: 10.1186/s12284-020-00410-7 (PMC7360007; doi:10.1186/s12284-020-00410-7)
Supplement: Supplementary file 2 — Additional file 2: Supplemental Figure 1. Technical validation of Aroma SNP1 across segregating F2:F3 breeding population. All genotypic classes showed clear clustering suitable for automated computer scoring with the exception of one line (indicated in grey). [file 12284_2020_410_MOESM2_ESM.pdf]

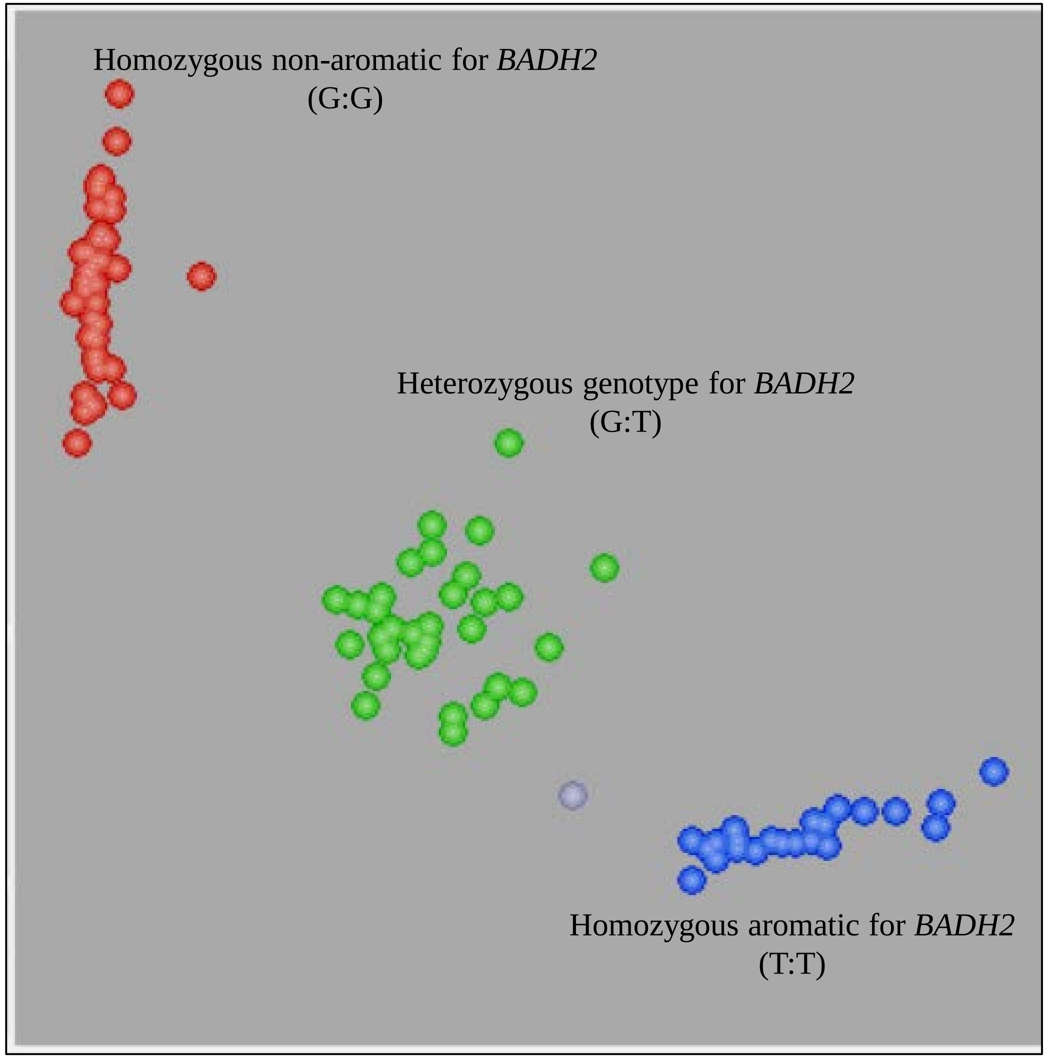

Supplemental Fig 1. Technical validation of Aroma SNP1 across segregating F2:F3 breeding population. All genotypic classes showed clear clustering suitable for automated computer scoring with the exception of one line (indicated in grey).
